# Cycling for Transportation in Sao Paulo City: Associations with Bike Paths, Train and Subway Stations

**DOI:** 10.3390/ijerph15040562

**Published:** 2018-03-21

**Authors:** Alex Antonio Florindo, Ligia Vizeu Barrozo, Gavin Turrell, João Paulo dos Anjos Souza Barbosa, William Cabral-Miranda, Chester Luiz Galvão Cesar, Moisés Goldbaum

**Affiliations:** 1 School of Arts, Sciences and Humanities, University of Sao Paulo, Sao Paulo City 03828-000, Brazil; 2 Graduate Program in Nutrition in Public Health, Department of Nutrition, School of Public Health, University of Sao Paulo, Sao Paulo City 01246-904, Brazil; jpdosanjos@usp.br; 3 Department of Geography, School of Philosophy, Literature and Human Sciences, University of Sao Paulo, Sao Paulo City 05508-080, Brazil; lija@usp.br (L.V.B.); williamcabral@usp.br (W.C.-M.); 4 Institute for Health and Ageing, Australian Catholic University, Melbourne, VIC 3065, Australia; gavin.turrell@acu.edu.au; 5 Department of Epidemiology, School of Public Health, University of Sao Paulo, Sao Paulo City 01246-904, Brazil; clcesar@usp.br; 6 Department of Preventive Medicine, School of Medicine, University of Sao Paulo, Sao Paulo City 01246-903, Brazil; mgoldbau@usp.br

**Keywords:** cycling for transportation, bike paths, train stations, subway stations, adults, Brazil

## Abstract

Cities that support cycling for transportation reap many public health benefits. However, the prevalence of this mode of transportation is low in Latin American countries and the association with facilities such as bike paths and train/subway stations have not been clarified. We conducted a cross-sectional analysis of the relationship between bike paths, train/subway stations and cycling for transportation in adults from the city of Sao Paulo. We used data from the Sao Paulo Health Survey (*n* = 3145). Cycling for transportation was evaluated by a questionnaire and bike paths and train/subway stations were geocoded using the geographic coordinates of the adults’ residential addresses in 1500-m buffers. We used multilevel logistic regression, taking account of clustering by census tract and households. The prevalence of cycling for transportation was low (5.1%), and was more prevalent in males, singles, those active in leisure time, and in people with bicycle ownership in their family. Cycling for transportation was associated with bike paths up to a distance of 500 m from residences (OR (Odds Ratio) = 2.54, 95% CI (Confidence interval) 1.16–5.54) and with the presence of train/subway stations for distances >500 m from residences (OR = 2.07, 95% CI 1.10–3.86). These results are important to support policies to improve cycling for transportation in megacities such as Sao Paulo.

## 1. Introduction

Cities that promote and support cycling for transportation reap many public health benefits for their populations, such as reduced risk for chronic disease, lower rates of overweight and obesity, fewer traffic accidents and injuries, and lower levels of air pollution [1,2,3,4,5]. However, increasing the population prevalence of cycling for transportation is a big challenge for many countries. In Latin American cities, for example, less than 10% of the adult population use a bicycle for transport [6]. This contrasts with European countries such as the Netherlands and Denmark, where more than 25% of transport-related trips are undertaken by bicycle [7].

Cycling for transportation is most prevalent in men and in people of middle-age and young adults [3,8,9,10,11,12,13,14]. Studies from high-income countries show that built and social environments as well as associated policies are very important in terms of increasing the use of the bicycle for transportation purposes. Two reviews showed that bike paths, a safe riding environment, integration of the bicycle with other forms of transportation, bike parking, bicycle ownership, and interventions based on education and mass media are important factors for increasing the use of this mode of transportation [15,16]. Empirical studies show that access to bike paths close to residences is associated with cycling for transportation [8,10,11,12,17,18,19]. However, knowledge of the factors that explain the use of cycling in Latin American countries is limited. For example, a review of cycling for transportation in Curitiba (Brazil), Santiago (Chile), and Bogota (Colombia) recommended that policy-makers increase bike path availability and accessibility as a way of promoting active transportation [6]. However, a study conducted in Curitiba found no significant association between the presence of bike paths in 500-m buffers with cycling for transportation [20]. This is important because some megacities in upper-middle income countries such as Brazil and Colombia are increasing the length of bike paths to promote and support cycling. Sao Paulo (Southeastern, Brazil) is a good example, because this city has more than 400 km of bike paths. In addition, 4.7 million people use the subway daily for transportation on weekdays [21]. In this case, it is important to investigate if bike paths and train or subway stations in Sao Paulo are associated with cycling for transportation. Therefore, the aims of this study were: (1) to identify the profile of adults that use cycling for transportation in Sao Paulo city; (2) to assess whether the presence of train or subway stations is associated with cycling for transportation; (3) to examine if the distance of bike paths from residences is associated with cycling for transportation; and (4) to assess if the mix of the presence of train or subway stations and the distance of bike paths from residences is associated with cycling for transportation.

## 2. Material and Methods

### 2.1. Sao Paulo Health Survey

**Figure 1:**
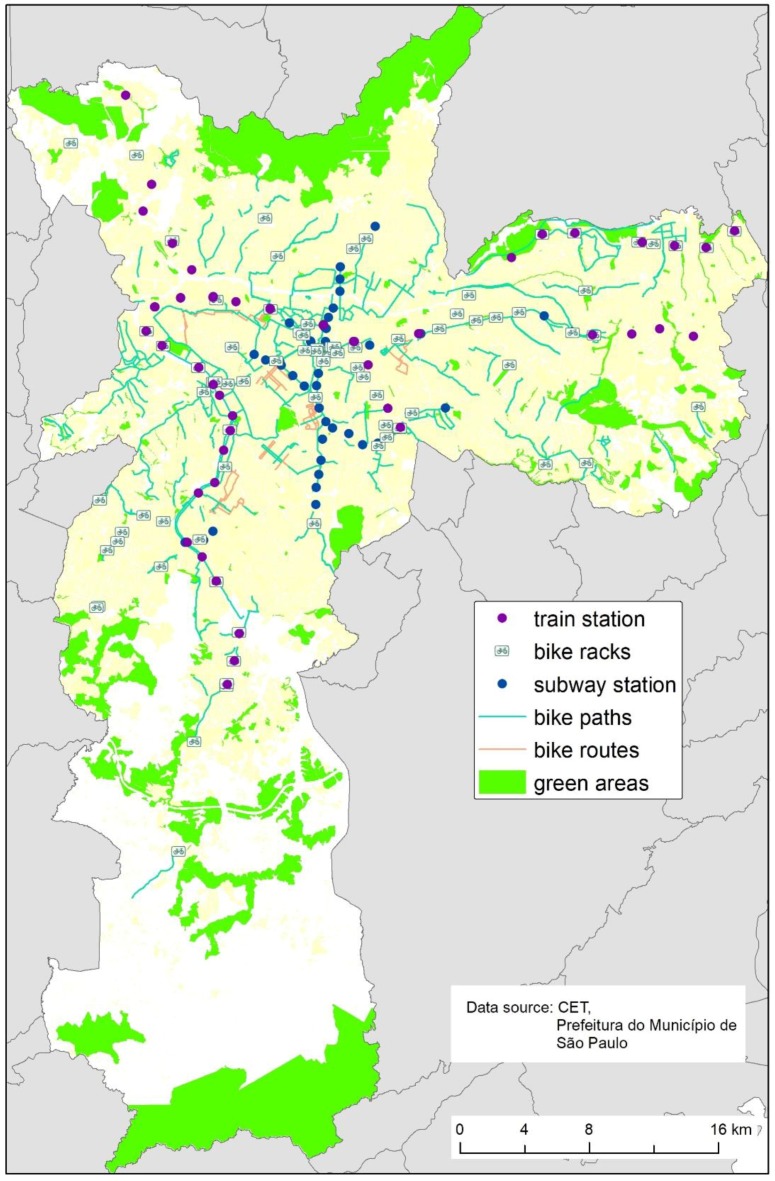
Map of bike paths, bike lanes, bike racks, train and subway stations in Sao Paulo, 2017.

Sao Paulo had a population of 12,038,175 inhabitants living in 1521.11 km^2^ in 2017. The city is divided in 96 districts, 32 administrations, concentrates 11% of gross national product from Brazil, and is one of the 10 most populated cities in world. Sao Paulo currently has 468 km of bike paths, 80.4 km of subway, and 71 stations (Figure 1) [21,22].

The sample for the Sao Paulo Health Survey included 3145 adults (aged 18 years or more) who were interviewed in their homes and had their residential addresses’ geocoded. The Sao Paulo Health Survey is a cross-sectional study based on a representative sample of adults who lived in Sao Paulo city in 2014 and 2015. Details about the sampling and geocoding process are described elsewhere [23].

The Ethics Committee of the School of Arts, Sciences, and Humanities at the University of Sao Paulo approved this study (process number 55846116.6.0000.5390).

### 2.2. Outcome Variable

The outcome was cycling for transportation measured using the International Physical Activity Questionnaire (IPAQ) long version. The IPAQ has been validated and used in the Brazilian adult population [24], and this module was standardized to capture the frequency (times per week) and duration (minutes per day) of cycling for transportation. In this study we focus only on the binary outcome: the use of a bicycle for transportation in a normal week (yes or no).

### 2.3. Covariates

The covariates used in this study were: sex (men, women), age (18–29 years, 30–39 years, 40–49 years, 50–59 years, and 60 years or older), education (incomplete elementary school, elementary to incomplete high school, complete high school, undergraduate incomplete to complete), marital status (married/de facto, single, divorced/separated/widowed), physical activity in leisure-time measured using the IPAQ long (≥150 min per week, <150 min per week), body mass index based on self-reported height and weight (≥30 kg/m^2^, <30 kg/m^2^), smoking (yes or no), self-report of health (good/very good/excellent, regular/bad/very bad), employment situation (work: yes or no), car or motorcycle ownership (yes or no), bicycle ownership (yes or no), and length of living in the residence (up to 1 year, between 1 and 5 years, >5 years).

### 2.4. Built Environment Variables

We used a georeferenced street dataset from Sao Paulo to obtain bike paths as well as train and subway stations [22]. We used a Geographic Information System to delineate radial buffers of 1500 m based on the geographic coordinates of the adults’ residential addresses. This distance is based on the distance that people can access within 15 min of walking [25]. We used Arc Map software (version 10.3, Redlands, CA, USA).

Within each buffer we recorded: (1) the presence or absence of train or subway stations; (2) the distance of bike paths from the participants’ residences.

### 2.5. Statistical Analysis

We calculated the prevalence ratio for cycling for transportation according to social, demographics, health, and work characteristics. We used Poisson regression and complex sample design according to the census tract (primary unit of sample) in five health areas in Sao Paulo (strata), and the sample weight. This weight took into account the design effect, post-stratification by sex and age, and the non-response rate. For these analyses we used Stata (version SE 12.1, StataCorp, College Station, TX, USA).

To examine the relationship between the built environment and cycling for transportation, we conducted multilevel logistic regression. We used three independent variables: (1) the presence of train or subway stations; (2) the distance of bike paths from residences; and (3) the mix of the presence of train or subway stations and the distance of bike paths from residences. We used the distance of up to 500 m and above 500 m from participants’ residences to compare with places without facilities [23]. The modeling was undertaken in two stages: Model 1 took into account clustering by census tract and household, and was adjusted for sex and age; Model 2 used all variables in Model 1 plus adjustment for education, the time that people lived at the same residence, and the area of residence (North, Midwest, Southeast, South, East).

For multilevel analyses of this article, we had 34 missing data points and we worked with *n* = 3111 people in 2224 residences and 149 census tracts. All analyses were undertaken using the xtmelogit command in Stata (version SE 12.1, StataCorp).

## 3. Results

The overall prevalence of cycling for transportation was 5.1% (95% CI 4.0–6.2). The prevalence ratio was high for males, singles, those active in leisure time, and for people with bicycle ownership in their family (Table 1).

**Figure 2:**
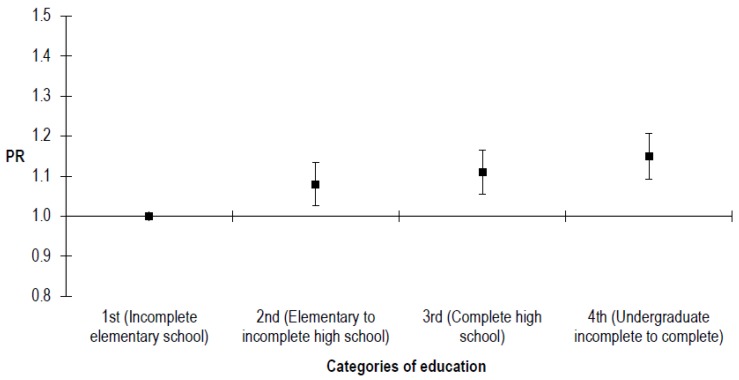
Prevalence ratio (PR) for bicycle ownership according to education and adjusted by age and sex.

Bicycle ownership in families was more prevalent according to educational level (*p*-trend < 0.001). The prevalence ratio was 1.15 (95% CI 1.09–1.23) for people that had incomplete undergraduate studies than people had only incomplete elementary school (Figure 2).

Just under one-third of people had bike paths or train or subway stations within 500 m of their residence (Table 2). Only a quarter of people had train or subway stations within 1500-m buffers (Table 2).

After adjusting for the covariates, the odds of cycling for transportation were 154% higher if there was a bike path within 500 m of a person’s residence (Table 3).

The presence of train or subway stations was associated with cycling for transportation in distances above 500 m from residences: for people living in buffers with these facilities the odds of cycling for transport were 107% higher, independent of sex, age, education, places where people live, and length of time living in the same residence (Table 3). 

There was no association found between cycling for transport with the mix of train or subway station and bike paths in the same buffers (Table 3). 

## 4. Discussion

This study showed that the presence of bike paths within 500 m of residences and the presence of train or subway stations within 1500 m of residences were associated with the prevalence of cycling for transportation in adults living in Sao Paulo. However, the prevalence of cycling for transportation was generally low, although it was higher in males, singles, those active in leisure time, and in people with bicycle ownership in their family. 

An international review showed that most of the studies found a positive significant association between bike paths and cycling in high-income countries [15]. Dill and Carr found that with a greater amount of bike paths, the prevalence of commuting by bicycle was higher in 35 cities in the United States [19]. A study conducted in Brisbane, Australia, showed that people living in neighborhoods with more kilometers of bike paths had a significantly greater likelihood of cycling for transport [26]. Bike paths are very important to cycling in high-income countries, and in the Netherlands and Germany the investment to improve these facilities paths started in the 1970s [7]. However, in Latin American countries, these actions and facilities have been implemented more recently, with the wider creation of bike paths in Sao Paulo beginning in 2014. The discussion now is about possible effects of these facilities on the use of cycling for commuting. We found a significant likelihood of cycling for transportation for people who lived in residences within 500 m of bike paths. However, other studies conducted in Brazil found results different from those reported here. Hino et al. did not find a significant association between the presence or the length of bike paths and cycling for transport in adults who lived in Curitiba, south of Brazil [20]. A review that discussed transport and health in Chile, Brazil, and Colombia, however, recommended the adoption of bike paths to improve cycle commuting in cities in Latin American countries [6]. We need more cross-sectional and longitudinal studies in Latin American cities and in other megacities from upper- and middle-income countries to verify the contribution of bike paths because many factors may influence the use of the bicycle for transportation purposes.

We did not find a significant association between cycling for transportation and bike paths at distances greater than 500 m from residences. A study conducted with adults from Arlington, USA, showed that for each increase of 0.25 miles (~400 m) in the distance from residences to trails and bike paths, the likelihood of cycling decreases [11]. Another study conducted with adults from King County, Washington, USA, showed that people who lived in places that had bike paths or trails within 0.5 m of their residence (~800 m) had a significant likelihood of commuting by bicycle [8]. In cities like Sao Paulo, it can be very difficult to access bike paths located long distances from residences because there exist many barriers such vehicle traffic and streets on which it is dangerous to cycle. Some areas of Sao Paulo also present steep hills, which may discourage cycling. In addition, studies show that safety perception is associated with cycle commuting in different populations [12,15,27], and the presence of bike paths in neighborhoods is associated with a better safety perception of bicycle use [28].

We found a significant likelihood of cycling for transportation associated with the presence of train or subway stations, but only for distances greater than 500 m from residences. In addition, there was no association found between cycling for transport and the mix of bike paths and train or subway stations in the same buffers. The odds ratio was not improved with the inclusion of train and subway stations in the models of bike paths within 500 m of residences, which were significant to the increased prevalence of cycling for transportation. We had a low number of train and subway stations located within 500 m of residences (<5%) as well as a low incidence of bike paths and train or subway stations in the same buffers where people lived (<5% within 500 m and <15% above 500 m). Probably as a consequence of this, we did not find a significant association with these factors. We did not find other studies that examined the relationship between the presence of train and subway stations in different buffer sizes and cycle commuting. However, a study conducted with adults from Brisbane, Australia, found that people who perceived transport destinations to be within 20 min of walking from their residences had a significant likelihood of cycling for transportation [29].

In addition, other facilities are important to increase cycling for transportation. Winters et al. showed that not only bike paths were important for cycling for transport, but also hills, destinations, and connectivity [30]. The authors examined the bike score that was based on bike paths, hills, and destinations in census tracts and showed that this score was positively associated with the cycling commuting in 24 cities in the USA and Canada. A study conducted in Stockholm, Sweden, showed that factors such as aesthetics, greenery, car traffic, and noise were important factors affecting bikeability [31]. However, in Curitiba, Brazil, Hino et al. showed an inverse association between land use mix and cycling for transportation in adults [20]. Therefore, we not only need to increase the number of bike paths, but also better integrate cycling facilities with train and subway stations [15]. For example, people cannot transport bicycles in the subway in Sao Paulo during weekdays until 8:30 pm., there are a only few bicycle parking locations in operation [21], and the bike-share system has been found to be unstable and insufficient for the city in last two years.

Another important question is about bicycle ownership, which was found to be more likely in people with better education. A study conducted with adults who lived in Seattle, Baltimore, and Washington, USA, showed that bicycle ownership was positively associated with education level [12]. Sá et al. showed a significant decrease in the prevalence of cycling in Sao Paulo between 2007 to 2012 in low-income adults as well as an increase of cycling prevalence in high-income adults in the same period [3]. We showed that the prevalence of cycling for transportation was more common in people who had bicycle ownership in their family. These results are important because they show that we need more policies to improve the population’s capacity to purchase bicycles in Brazil and improve bicycle-share programs with low costs.

We found a low prevalence of cycling for transportation in the adult population in Sao Paulo (~5%). Surveys conducted with adults who lived in three cities from Brazil showed that the prevalence of cycling for transport varied between 8.8% in Vitoria (Southeast) to 16.6% in Recife (Northeast), and 13.3% for the total population [32]. A trend-study conducted with adults from Sao Paulo showed that there was an increased number of cyclists from 1997 (3.9 per 1000 people) to 2007 (6.3 per 1000 people) but a decrease in 2012 (5.4 per 1000 people) [3]. Compared with European countries that are cycling-friendly such as the Netherlands, where 27% of trips are made by bicycle, Brazil is very far from achieving widespread cycling for transportation [7]. Only in Rio Claro (a small city in Sao Paulo State) did a survey find 28.3% transport cycling prevalence. The authors argued that this city is ideal for cyclists because it is a small city with a flat topography with many kilometers of bike paths and bike lanes. Moreover, this city has poor public transportation with few services that are expensive for the population [14].

The prevalence of cycling for transportation was higher in males and singles that are generally younger adults. This profile is similar to studies conducted in Brazil, Australia, Canada, and the United States [8,9,11,32,33,34]. Another interesting finding was that cyclists were more active in leisure time. These results are consistent with the literature, which showed that cycling for transportation is a healthy activity. Studies have shown that active transportation and cycling for transportation contribute to increased physical activity and life expectancy and are associated with lower odds of obesity, hypertension, and high triglycerides [4,5,35]. In addition, a study published by Tainio et al. showed that the benefits of cycling in Sao Paulo overcome the risks of air pollution exposure [36]. Therefore, these results are important because an increase in cycling for transportation can contribute to increasing physical activity, for health promotion, and for disease prevention.

In addition, we think that increasing the prevalence of cycling for transportation is much more complex than merely building bike paths or train/subway stations. We need intervention programs such as media campaigns and educational programs in communities and schools [15]. For example, surveys conducted with Colombian adults in Bogota showed that people who participated in the community-based “Ciclovia Program” were significantly more likely to cycle for transportation [37]. Another study conducted in Cali, Colombia, showed that adults’ participation in the “Ciclovia Program” was associated positively with the presence of a “Ciclovia corridor”, which are streets closed for cars and open for walking or cycling on weekends and public holidays [38]. The longitudinal study “Cycling Connecting Communities” conducted with adults in Sydney, Australia, based on a social marketing framework and behavior change theories, found that after two years people who received the intervention had a significant likelihood of using bike paths [28]. In addition, other variables are associated with cycling for transportation such as self-efficacy, physical activity habit, attitude, and social support [29,38]. Finally, we need better facilities in subways and train stations, such as permission to travel with bicycles, bicycle parking, and bicycle-share programs across the cities. 

This study has a number of limitations. Firstly, as the study was cross-sectional we cannot be sure if interviews were conducted after the implementation of bike paths, because the interviews were conducted from August 2014 to December to 2015, during which time many kilometers of bike paths were built in Sao Paulo. A longitudinal analysis like that conducted in England [39] may provide better evidence about the possible influences of bike paths on bicycle use for people living in Sao Paulo. Secondly, we worked with radial buffers that may introduce measurement error [40]. Future analysis with network buffers around residences should be compared with radial buffers. Thirdly, we did not control for self-selection in the neighborhood [41]; people who are cyclists may choose to live in neighborhoods with better bike paths and subway or train station facilities.

## 5. Conclusions

An increase in cycling for transportation can contribute to improved physical activity, reduced cardiovascular and respiratory disease, reduced air pollution, and improved public health and well-being in big cities [1,2,4,5,35,42]. We showed that bike paths close to residences (within 500 m) as well as access to train or subway stations (1500-m buffers) are factors that can increase cycling for transportation in Sao Paulo. However, the prevalence of cycling for transportation is low in Sao Paulo city (5.1%) and increasing this activity is not an easy task because active transport has multiple determinants. We need an increase in bike paths and a better integration of cycling with train and subway stations, including better and more secure facilities such as bike parking in addition to bicycle transportation in subway and train stations. Moreover, people need more opportunities to own a bicycle, a more extensively developed bike-share program, and community-based interventions to demonstrate the opportunities and benefits of this type of transportation for different populations.

## Figures and Tables

**Table 1:** Descriptive characteristics and prevalence ratio for cycling for transportation in adults from Sao Paulo, 2015.

Variables	Sample Characteristics (%) *	Prevalence Ratio (95 %CI) **
Sex (*n* = 3145)		
Female	53.5	1.00
Male	46.5	**4.59 (2.82–7.46) ^#^**
Age group (*n* = 3145)		
18–29 years	24.5	1.00
30–39 years	22.8	1.18 (0.74–1.89)
40–49 years	18.7	1.37 (0.76–2.45)
50–59 years	15.8	0.66 (0.33–1.34)
60 years or older	18.2	0.63 (0.29–1.41)
Education (*n* = 3145)		
Incomplete elementary school	17.4	1.00
Elementary to incomplete high school	24.0	0.93 (0.50–1.75)
Complete high school	29.0	0.89 (0.49–1.59)
Undergraduate incomplete to complete	29.6	1.00 (0.51–1.98)
Marital Status (*n* = 3136)		
Married or de facto	56.9	1.00
Single	28.3	**1.64 (1.04–2.59) ^#^**
Divorced, separated, or widowed	14.8	1.33 (0.74–2.36)
Physical activity in leisure time (*n* = 3141)		
<150 min per week	78.0	1.00
≥150 min per week	22.0	**1.59 (1.11–2.28) ^#^**
Obesity (*n* = 3077)		
Body mass index ≥ 30 kg/m^2^	79.0	1.00
Body mass index < 30 kg/m^2^	21.0	1.28 (0.73–2.24)
Smoking (*n* = 3142)		
No	82.6	1.00
Yes	17.4	1.10 (0.68–1.79)
Self-report of health (*n* = 3141)		
Regular/bad/very bad	27.2	1.00
Good/very good/excellent	72.8	1.22 (0.81–1.84)
Employees (*n* = 3100)		
No	34.9	1.00
Yes	65.1	1.50 (0.99–2.27)
Car or motorcycle ownership (*n* = 3145)		
No	42.8	1.00
Yes	57.2	0.77 (0.52–1.15)
Bicycle ownership (*n* = 3052)		
No	68.3	1.00
Yes	31.7	**4.48 (2.95–6.79) ^#^**
Time in the same residence (*n* = 3111)		
Up to 1 year	12.4	1.00
Between 1 and 5 years	21.0	0.90 (0.49–1.67)
More than 5 years	66.6	0.73 (0.42–1.28)

CI: confidence interval; * Weighted percentage; ** Adjusted by all variables in Table 1; **^#^** *p* < 0.05.

**Table 2:** Distribution of facilities according to participants’ residence.

Facilities in 1500-m Buffers	% of People with Bike Paths *	% of People with Train/Subway Stations **	% of People with Bike Paths and Train/Subway Stations in the Same Buffers ***
None	30.9	70.9	80.8
Up to 500 m	29.3	4.7	4.4
Bewteen 500 m and 1500 m	39.8	24.4	14.8

* Distance from the participants’ residence; ** only the presence (yes or not); *** According to distances between bike paths from residences and the presence of train or subway stations in these distances.

**Table 3:** Results of the multilevel model for the association between cycling for transportation with bike paths and train or subway stations.

Bike Paths	Model 1	Model 2
	OR (95% CI) *	OR (95% CI) **
Bike paths only		
None	1	1
Up to 500 m	**2.11 (1.04–4.27) ^#^**	**2.54 (1.16–5.54) ^#^**
Between 500 m and 1500 m	1.14 (0.59–2.21)	1.62 (0.78–3.36)
Train or subway stations only		
None	1	1
Up to 500 m	1.30 (0.36–4.61)	1.26 (0.33–4.74)
Between 500 m and 1500 m	**1.87 (1.01–3.45) ^#^**	**2.07 (1.10–3.86) ^#^**
Bike paths and train or subway stations ***		
None	1	1
Up to 500 m	0.78 (0.20–3.04)	0.72 (0.17–3.00)
Between 500 m and 1500 m	0.88 (0.41–1.88)	1.15 (0.54–2.48)

OR: Odds ratio; CI: confidence interval; * Model 1: adjusted by sex, age; ** Model 2: adjusted by variables in Model 1 plus education, place where people lived in Sao Paulo, and the length of time that they lived in the same residence; *** in the same buffers; **^#^** *p* < 0.05.
